# Pharmacogenomics of Monoclonal Antibodies for the Treatment of Rheumatoid Arthritis

**DOI:** 10.3390/jpm12081265

**Published:** 2022-07-31

**Authors:** Sung Ho Lim, Khangyoo Kim, Chang-Ik Choi

**Affiliations:** 1 Integrated Research Institute for Drug Development, College of Pharmacy, Dongguk University-Seoul, Goyang 10326, Korea; 93sho617@naver.com; 2 College of Pharmacy, Dongguk University-Seoul, Goyang 10326, Korea; khangyoo@naver.com

**Keywords:** pharmacogenomics, precision medicine, rheumatoid arthritis, monoclonal antibody, genetic polymorphism

## Abstract

Precision medicine refers to a highly individualized and personalized approach to patient care. Pharmacogenomics is the study of how an individual’s genomic profile affects their drug response, enabling stable and effective drug selection, minimizing side effects, and maximizing therapeutic efficacy. Rheumatoid arthritis (RA) is an autoimmune disease that causes chronic inflammation in the joints. It mainly starts in peripheral joints, such as the hands and feet, and progresses to large joints, which causes joint deformation and bone damage due to inflammation of the synovial membrane. Here, we review various pharmacogenetic studies investigating the association between clinical response to monoclonal antibody therapy and their target genetic polymorphisms. Numerous papers have reported that some single nucleotide polymorphisms (SNPs) are related to the therapeutic response of several monoclonal antibody drugs including adalimumab, infliximab, rituximab, and tocilizumab, which target tumor necrosis factor (TNF), CD20 of B-cells, and interleukin (IL)-6. Additionally, there are some pharmacogenomic studies reporting on the association between the clinical response of monoclonal antibodies having various mechanisms, such as IL-1, IL-17, IL-23, granulocyte-macrophage colony-stimulating factor (GM-CSF) and the receptor activator of nuclear factor-kappa B (RANK) inhibition. Biological therapies are currently prescribed on a “trial and error” basis for RA patients. If appropriate drug treatment is not started early, joints may deform, and long-term treatment outcomes may worsen. Pharmacogenomic approaches that predict therapeutic responses for RA patients have the potential to significantly improve patient quality of life and reduce treatment costs.

## 1. Introduction

Precision medicine is defined as the diagnosis and treatment tailored to the patient based on their genotype, biomarkers, phenotype, or psychosocial characteristics to minimize unnecessary adverse events and improve clinical outcomes [1,2]. Medicines manufactured with the “one-size-fits-all” approach are effective in some patients and have no or minor side effects, while others are ineffective and have strong side effects [3]. To overcome the limitations of this one-size-fits-all framework in which all individuals presenting with some constellation of symptoms receive similar treatment, the diagnosis and management of various diseases are undergoing a paradigm shift to a personalized approach that prevents or treats diseases by considering each patient’s characteristics [4]. The paradigm shift towards precision medicine enables accurate disease prediction and prevention, reduces individual side effects and inefficient prescriptions, and provides safer diagnoses and treatments [5]. 

Over the past few decades, human genetics research has been fueled by cutting-edge sequencing technologies that lead to a deeper understanding of the relationship between genetic variation and health [6]. Pharmacogenomics is an emerging application to adjust drug selection and dosage considering a patient’s genetic characteristics [7]. It is one aspect of clinical genomics that will have the earliest and broadest clinical implementation with the potential to impact the treatment of all patients [8]. Although several pharmacogenetic guidelines have been reported by international scientific consortia in recent years, the application of pharmacogenomics in clinical care is still limited. Various major barriers have been identified from basic pharmacogenomics research to implementation, and many coordinated international efforts are underway to overcome them [9]. Studies of previously neglected rare genetic variants and validation of their function and clinical impact through preclinical models are essential to advance pharmacogenetic knowledge. 

Another technological advancement related to precision medicine is the development of “biopharmaceuticals”. The biopharmaceutical market is advancing faster than all pharmaceutical markets, with innovations such as immunotherapy, antibody-drug conjugates, and gene therapy [10]. Biopharmaceuticals rarely cause side effects and show high specificity and activity compared to conventional drugs [11]. Their properties could provide targeted therapies rather than symptomatic treatments, accelerating treatment for conditions that cannot be treated with conventional synthetic drugs. [12]. Among biopharmaceuticals, monoclonal antibodies are the most profitable and are used to cure a variety of diseases, including autoimmune diseases, angiogenesis-related diseases, cardiovascular diseases, inflammatory diseases, and cancer [13]. The top 10 best-selling biopharmaceuticals in 2017 contained eight Abs. Among this list, the mAb adalimumab (ADA), a tumor necrosis factor-alpha (TNF-α) inhibitor used to treat rheumatoid arthritis and related disorders, was the most profitable product each year, generating global sales of approximately USD 62.6 billion between 2014 and 2017 [14]. The number of newly registered monoclonal antibodies is predicted to steadily increase and dominate the biopharmaceutical market [10].

Rheumatoid arthritis (RA) is an autoimmune disease that causes chronic inflammation of the joints throughout the body. Mainly, the destruction of articular cartilage and bone damage proceed due to persistent synovitis and infiltration of immune cells in the peripheral joints of the hands and feet [15]. As the disease progresses, chronic pain caused by functional impairment and joint deformation causes physical disability, reduced quality of life, and cardiovascular and other comorbidities [16]. Nonsteroidal anti-inflammatory drugs (NSAIDs) used to treat RA do not interfere with joint damage and therefore do not cure the disease. Glucocorticoids provide rapid symptom and disease correction but are associated with serious long-term side effects [17]. Disease-modifying antirheumatic drugs (DMARDs) used to coordinate disease progression through anti-inflammatory and immunomodulatory actions are key to RA treatment [18]. Commonly used primary conventional synthetic DMARDs to treat RA include methotrexate, hydroxychloroquine, and sulfasalazine. In case of ineffectiveness, leflunomide or tacrolimus (calcineurin inhibitor) are used. In the past, immunosuppressive agents such as azathioprine or cyclosporine, and parenteral gold, penicillamine, and bucillamine were often used for general autoimmune diseases, but they are now rarely prescribed due to the development of more effective and safer drugs [19]. When faced with the limitation that a sufficient therapeutic effect cannot be obtained with the above therapeutic agents, biological DMARDs (TNF-α inhibitors, B-cell modulation, and interleukin (IL)-6R blockade) or targeted DMARDs (Janus kinase inhibitors) may be used alone or together with existing conventional synthetic DMARDs [20]. Most biological DMARDs exhibit enhanced efficacy when combined with other conventional synthetic DMARDs [21]. These drugs are designed to target inflammatory molecules, cells, and pathways that cause tissue damage in patients with RA [22]. In this study, we summarized the pharmacogenetic studies of mAb drugs among biological DMARDs of RA.

## 2. Adalimumab

The discovery of the role of certain cytokines, especially TNF-α, in the pathogenesis of RA has dramatically changed disease treatment [23]. TNF-α is one of the major mediators abundantly expressed in the synovial fluid and synovium of patients with RA [24]. They modulate immune responses that have a powerful impact on cellular and humoral immunity [25]. For example, TNF-α can induce both cartilage degradation and bone resorption, directly affecting osteocyte receptor activation of nuclear factor κB ligand (RANK-L) expression, and increasing osteoclast generation [26,27]. In addition, TNF-α contributes to the pathogenesis of RA by inducing the production of other inflammatory cytokines, such as interleukin (IL)-1β and IL-6, which promote inflammation in the synovial membrane and attract and accumulate leukocytes [28,29]. In the past several decades, these TNF-α blockers have shown excellent efficacy in improving the inflammation and joint destruction caused by RA, and angiogenesis inhibition has been observed in various clinical trials [30,31,32,33,34]. 

ADA was approved by the Food and Drug Administration (FDA) in 2002 as an anti-TNF-α drug made by producing a novel antibody protein by cloning “phage display” [35,36]. It is a fully recombinant human mAb that is structurally and functionally indistinguishable from naturally occurring human IgG1 [37]. ADA specifically binds to TNF-α and blocks interactions with p55 and p75 cell surface TNF receptors [38]. Many clinical trials indicate ADA’s efficacy and safety for RA. As a blockbuster drug, the largest number of biosimilars are already on the market or in development. Genetic polymorphisms associated with the ADA response are discussed in Table 1.

### 2.1. FCGR2A

Fc receptor IgG immunoglobulins (FCGRs) can bind to extracellular IgG and cause cell activation or inhibition [48]. Consequently, genetic mutations affecting the activity of FCGRs are likely to affect the therapeutic efficacy of immunoglobulin-based therapies such as anti-TNF drugs [49]. *FCGR2A*, which encodes an Fc receptor expressed in various immune cells but mainly in macrophages and dendritic cells, is correlated with anti-TNF treatment in RA treatment [50,51]. ADA, most commonly used to treat RA, has an IgG1 Fc portion capable of binding to FCGRs. Changes in Fc binding affinity may affect the response to these biological therapies [52]. 

Avila-Pedretti et al. [39] studied whether genetic mutations at the Fc receptor *FCGR2A* were associated with the response to the anti-TNF agent ADA. A total of 95 RA patients treated with ADA were included and genotyped for the FCGR2A polymorphism rs1081274. Response to ADA treatment was measured according to the European League Against Rheumatism response (EULAR) criteria. They measured disease activity scores (DAS) using 28 joint counts after 12 weeks of ADA treatment. There was a statistically significant association with the genotype frequency of the *FCGR2A* polymorphism rs1801274 according to EULAR extreme clinical response to ADA treatment (odds ratio (OR) = 2.54; confidence interval (CI)_95%_ = 1.9–5.4; *p* = 0.022). There have been several reports that RA patients positive for anti-cyclic citrullinated peptide (CCP) have a differential and stronger genetic background than anti-CCP-negative patients [53,54]. The association between the *FCGR2A* polymorphism rs1801274 and the response to ADA in RA patients in the anti-CCP positive group was investigated, and a significant relationship was confirmed (OR = 2.56; CI_95%_ = 1.18–5.54; *p* = 0.047).

### 2.2. DHX32

Additionally, Avila-Pedretti et al. [39] identified a microarray-based study in RA analyzing the transcriptome of synovial macrophages (*GSE49604*, *n* = 8 samples). They found *DEAH (Asp-Glu-Ala-His) box polypeptide 32 gene (DHX32)* was positively correlated with *FCGR2A* (average r^2^ = 0.93, *p* < 0.001). The *DHX32* gene encodes a putative RNA helicase and is involved in lymphocyte differentiation and activation [55,56]. In particular, RNA helicase is important for innate immune inactivation of viral RNA, which may contribute to the development of autoimmune diseases such as RA [57,58]. They selected a single nucleotide polymorphism (SNP) for *DHX32* and determined its association with clinical response to ADA treatment in the RA patient cohort. There was a significant association between the *DHX32* SNP rs12356233 and the clinical response to ADA (OR = 2.7; CI_95%_ = 1.3–5.61; *p* = 0.0064). Moreover, analysis of the anti-CCP positive group of RA patients still found a significant association with ADA (OR = 2.65.; CI_95%_ = 1.25–5.6; *p* = 0.0095).

### 2.3. RGS12

The regulator of *G protein signaling 12 gene (RGS12)*, which is specifically expressed in human osteoclasts, is essential for NF-κB inflammatory signaling and thus plays an important role in the progression of RA [59]. Deletion of *RGS12* attenuates inflammatory pain, which may be due to dysregulation of the COX2/PGE2 signaling pathway [60]. In another microarray-based study (*GSE1050*, *n* = 8 samples) in RA analyzing the transcriptome of synovial macrophages, Avila-Pedretti et al. [39] found that the *RGS12* gene was negatively correlated with *FCGR2A* gene expression (average r^2^ = −0.96, *p* < 0.001). The *RGS12* SNP rs4690093 confirmed a nominally significant association between the clinical response to ADA (OR = 0.4; CI_95%_ = 0.17–0.98; *p* = 0.04). This effect was similar when analyzing the anti-CCP positive group of RA patients (OR = 0.4; CI_95%_ = 0.16–0.99; *p* = 0.049).

### 2.4. IL-6

Dávila-Fajardo et al. [40] conducted a study validating the reported association of the *IL-6* −174G/C polymorphism rs1800795 with the anti-TNF response in an independent cohort of 199 RA patients. Patients were classified as good or moderate responders and non-responders to the EULAR criteria at 6, 12, 18, and 24 months after treatment with TNF-α inhibitors, including ADA. When comparing the allele frequencies of responders and non-responders, there were slightly more patients with the −174G/C *IL-6* polymorphism in the responder group compared with the non-responder group, but this was not statistically significant (*p* = 0.456). It was significantly associated with good or moderate EULAR response at 12, 18, and 24 months (OR = 2.93; CI_95%_ = 1.29–6.70; *p* = 0.011, OR = 5.17; CI_95%_ = 1.80–14.85; *p* = 2.27 × 10^−3^, and OR = 14.86; CI_95%_ = 2.91–75.91; *p* = 1.18 × 10^−3^, respectively). Their results confirm the role of the −174G/C *IL-6* polymorphism as a genetic predictive marker of responsiveness to anti-TNF therapy, including ADA.

### 2.5. PTPN22

Potter et al. [41] conducted a study to determine whether *PTPN22* genetic susceptibility mutations predicted response to ADA treatment in 68 RA patients. The difference in ADA treatment response between autoantibody-positive and -negative patients was observed, but there was no statistically significant difference using logistic regression analysis with the EULAR response criteria. No association between drug response and shared epitope or *PTPN22* R620W (C1858T) polymorphism was demonstrated in ADA (*p* > 0.05). In summary, although genetic factors are likely to contribute to treatment response, the well-established RA susceptibility loci, shared epitope, or *PTPN22* are not included.

### 2.6. TNF

Cuchacovich et al. [42] conducted the first study to investigate the effect of the −308 *TNF*-α polymorphism on the clinical response to ADA therapy in patients with RA. They genotyped 81 RA patients for the −308 *TNF*-α polymorphism by polymerase chain reaction-restriction fragment length polymorphism analysis. They then subdivided patients into two groups (G/A and G/G genotype), and clinical responses were compared using DAS28 at 8, 16, and 24 weeks. As a result, there were significantly more DAS28 responders in the G/G genotype group (88%) than in the G/A genotype group (68%) at week 24. Additionally, the average DAS28 improvement of the G/G genotype group was higher than that of the G/A genotype group at week 24 (2.5 and 1.8, respectively).

Seitz et al. [43] explored whether the −308 *TNF*-α promoter polymorphism affects the therapeutic response to ADA-containing anti-TNF-α therapy in 54 RA patients. The average improvement in DAS28 score after 24 weeks of anti-TNF-α therapy was 0.83 ± 0.15 in the A/A genotype group, 1.50 ± 0.16 in the A/G genotype group, and 2.72 ± 0.70 in the G/G genotype group (*p* < 0.0001). These results confirmed that RA patients with the *TNF*-α −308 G/G genotype responded better to anti-TNF-α treatment than RA patients with the A/A or A/G genotype.

O’Rielly et al. [44] performed a meta-analysis of *TNF*-α −308 G/A polymorphism rs1800629, predicting poor response to TNF-α inhibitors, including ADA, in RA patients. The results were extracted based on DAS28 or achieving at least ACR 20 response. The frequency of the A allele status was 119/531 (22%) in responders and 60/161 (37%) in non-responders of nine studies [42,43,61,62,63,64,65,66,67]. Regardless of the prescribed TNF-α inhibitors, the odds for the A allele state were significantly reduced in responders versus non-responders (OR = 0.43; CI_95%_ = 0.28–0.68; *p* = 0.000245). These results indicate that retention of −308 G/A polymorphism is predictive of a decreased response to TNF-α inhibitors, including ADA. 

However, not all patients respond well to TNF-α inhibitors, so Zeng et al. [45] additionally meta-analyzed 15 studies [42,43,46,61,62,63,64,65,66,67,68,69,70,71,72] with a total of 2,127 patients to evaluate the TNF-α promoter −308 G/A polymorphism. Results showed that RA patients with the G allele responded better to treatment (OR = 1.87; CI_95%_ = 1.26–2.79; *p* = 0.002). A separate meta-analysis of both studies showed that individuals with the A allele were associated with a weaker response to anti-TNF-α treatment with ADA than those with the G allele. In conclusion, individualized treatment can be suggested based on the *TNF*-α −308 G/A polymorphic genotype of RA patients.

Miceli-Richard et al. [46] conducted a study to determine whether *TNF*-α gene polymorphisms (−238A/G, −308A/G, and −857C/T) are genetic predictors of the clinical response to ADA in RA patients. A total of 380 patients were treated with ADA + methotrexate (*n* = 182), ADA + other DMARD (*n* = 96), or ADA alone (*n* = 102), and the results were recorded as DAS28, ACR response, and the Health Assessment Questionnaire-Disability Index at 12 weeks of treatment. Of these, 152 RA patients had an ACR50 response at 12 weeks, but the three tested *TNF*-α polymorphisms were not significantly related to the ACR50 response. The haplotype reconstruction of the *TNF*-α locus revealed the GGC haplotype (−238G/−308G/−857C) was present in more than 50% of patients and was significantly associated with a lower ACR50 response at 12 weeks only in the group treated with methotrexate and ADA (*p* = 0.0041). These findings indicate that a single *TNF*-α locus haplotype (−238G/−308G/−857C) present on both chromosomes is associated with a lower response to treatment with methotrexate and ADA in patients with RA.

### 2.7. TNFR2

Ongaro et al. [47] assessed whether the polymorphisms 676T>G in the *TNFR2* gene could affect the clinical response in 105 RA patients who received anti-TNFα therapy with ADA for one year according to the ACR criteria [73]. The percent improvement (20, 50, or 70%) of all efficacy variables included in the ACR score set represented patients with low, medium, and high response grades, respectively. They analyzed the adjusted ORs obtained by subdividing the number of patients by genotype by comparing ACR70 and ACR (50+20). As a result, after three and six months of ADA treatment, the risk of belonging to the ACR group for TG genotype patients was significantly increased by about three times compared to wild-type (TT) genotype patients (OR = 2.90; CI_95%_ = 0.95–8.89, and OR = 2.94; CI_95%_ = 1.15–7.56, respectively). Moreover, there was a significant increase in adjusted ORs after 3 and 12 months of ADA treatment when they compared ACR70 and ACR20 responders (OR = 3.78; CI_95%_ = 1.07–13.31, and OR = 4.30; CI_95%_ = 1.16–15.99, respectively). The OR values obtained for the ACR70 versus ACR (50+20) or ACR20 comparisons for the GG genotype were not significant. Because the total number of patients with the GG genotype was low (*n* = 8), individuals carrying the 676G allele were counted together (TG+GG). This group of patients was similar to patients with the TG genotype in both ACR70 versus ACR (50+20) or ACR20, with significant adjusted ORs after 12 months of treatment (OR = 3.50; CI_95%_ = 0.99–12.35). Therefore, the presence of one G allele tends to be a less responsive phenotype during anti-TNFα therapy involving ADA. In conclusion, the *TNFR2* 676 T/G genotype is associated with a low response to anti-TNFα therapy containing ADA, so it can be a useful genetic marker for predicting various response grades to anti-TNFα therapy.

## 3. Infliximab

Infliximab (IFX) is the first chimeric mAb (mouse/human) designed to block and neutralize TNF-α, a major inflammatory cytokine [74]. Since its introduction in 1998, it has revolutionized the induction and maintenance of treating RA and inflammatory bowel disease, namely Crohn’s disease and ulcerative colitis [75,76]. IFX reduces serum levels of inflammatory mediators and vascular endothelial growth factors as well as TNFα inhibition. It also reduces the expression of chemokines in synovial tissues and decreases lymphocyte migration to the joints in RA patients [77]. Genetic polymorphisms associated with IFX response are summarized in Table 2.

### 3.1. FCGR2A and FCGR3A

Avila-Pedretti et al. [39] also studied the association between the *FCGR2A* polymorphism rs1081274 and clinical response to IFX in a total of 126 RA patients treated with IFX. A comparison of the frequency of the *FCGR2A* polymorphism rs1801274 between a global cohort of IFX-treated responders and non-responders showed no statistically significant association between clinical response to *FCGR2A* polymorphism rs1801274 in patients treated with IFX (OR = 0.76; CI_95%_ = 0.44–1.32; *p* = 0.11). In contrast, the *FCGR2A* polymorphism rs1801274 was significantly associated with IFX response in the anti-CCP positive RA patients group (OR = 0.62; CI_95%_ = 0.32–1.22; *p* = 0.35).

Cañete et al. [78] evaluated the relationship between the functional SNP of the *FCGR2A* gene and response to IFX treatment in 91 RA patients. The *FCGR2A*-RR genotype is a risk factor for susceptibility to autoimmune diseases, as immune complexes are less efficiently cleared from the circulation in RA patients, leading to tissue damage [82]. RA patients with the low-affinity *FCGR2A*-RR genotype had a significantly better ACR20 response (RR: 60% and HH-RH: 33.3%; *p* = 0.035), while EULAR good and moderate responses only showed a significant trend after 30 weeks of IFX treatment (RR: 38.1% and HH-RH: 25.0%). They also showed an association between the low-affinity *FCGR2A*-RR genotype and decreased DAS28 with three parameters, including C-reactive protein (3v-CRP), using a linear model multivariate analysis. These results suggest that IFX can be eliminated less efficiently in RA patients with low-affinity variants than in RA patients with high-affinity variants (HH or RH). They also investigated the effect on the *FCGR3A* polymorphism and the clinical response to IFX in patients with RA. After six weeks of follow-up, the low affinity *FCGR3A* allele had a significantly higher ACR50 response (FF: 24.1% and VV-VF: 2.2%; *p* = 0.003) and EULAR good response rate (FF: 44.8% and VV-VF: 22.9%; *p* = 0.040). Changes in DAS28 3v-CRP during follow-up were similar to those found in ACR and EULAR responses. In conclusion, the response to IFX treatment in RA patients is affected by the *FCGR3A* genotype.

### 3.2. RGS12

Avila-Pedretti G et al. [39] identified RGS12 as having a strong correlation with *FCGR2A* expression. Like the association between *FCGR2A* and clinical response of IFX, they found a nominally significant association between *RGS12* SNP rs2857859 and response to IFX in anti-CCP positive RA patients (OR = 0.4; CI_95%_ = 0.17–0.99; uncorrected *p* = 0.042). This association was not significant after several test corrections.

### 3.3. PTPN22

Potter C et al. [41] evaluated the role of the *PTPN22* R620W (C1858T) polymorphism as a predictor of ADA as well as IFX treatment outcomes in 296 patients with RA. Compared to rheumatoid factor (RF)-negative patients, RF-positive patients showed significantly less improvement in DAS28 values after anti-TNF therapy, including IFX as well as ADA and etanercept (OR =−0.48; CI_95%_ = −0.87–0.08; *p* = 0.018). Moreover, patients positive for anti-CCP antibody showed less improved DAS28 values compared to anti-CCP negative patients (OR = −0.39; CI_95%_ = −0.71–0.07; *p* = 0.017). Additionally, the effects of RF and anti-CCP antibodies were evaluated using multivariate linear regression combining both antibodies with previously known predictors, such as baseline HAQ and concurrent DMARD therapy and gender. As a result, RF and anti-CCP positivity did not better predict the response to anti-TNF therapy, and there was no association between these two factors and drug response (RF: R^2^ = 0.17; anti-CCP: R^2^ = 0.17; RF + anti-CCP: R^2^ = 0.17). Finally, they performed a linear regression including the interaction between drug type and autoantibody status to confirm that the predictive effects of RF and anti-CCP antibodies on IFX response were the same. The results showed that the effects of RF and anti-CCP antibodies were demonstrated in RA patients treated with IFX, but the effects were not statistically significant between the two drug types. As mentioned previously, an association between the tested anti-TNF therapies, including ADA, IFX, etanercept, and the *PTPN22* R620W (C1858T) polymorphism, has not been demonstrated (OR = −0.11; CI_95%_ = −0.36–0.15; *p* = 0.41). Therefore, the presence of these antibodies accounts for only a small part of the change in treatment response. *PTPN22* does not affect IFX efficacy.

### 3.4. MHC

Martinez et al. [79]. studied the association of major histocompatibility complex (MHC) polymorphisms with clinical response to IFX. They genotyped *HLA-DRB1*, *HLA-DQA1*, *HLA-DQB1*, MHC class I chain-related gene A (MICA) transmembrane polymorphism alleles, and TNFa-e, D6S273, HLA–B–associated transcript 2 (BAT2), and D6S2223 microsatellites in 78 RA patients who received IFX treatment. A control sample from 323 healthy individuals was also included to detect the linkage disequilibrium between maker pairs. The results showed no single allele associated with IFX response, including the TNFa/b microsatellite allele linked to the *TNF* promoter polymorphism. The frequency of the TNFa11;b4 mini-haplotype was increased (41% versus 16% in non-responders, *p* = 0.01) and that of the D6S273_3 allele was decreased in the responders (32%) versus non-responders (56%, *p* = 0.04). The D6S273_4/BAT2_2 pair was observed more frequently in the responders (46% versus 11% in non-responders, *p* = 0.001). This allele pair was only associated with the responder group when compared to the control group (46% in responders versus 17% in controls, *p* = 0.00002). They did not identify statistically significant differences in the frequency of MICA and D6S2223 polymorphisms and *HLA-DRB1*, *HLA-DQA1*, and *HLA-DQB1* alleles in responders and non-responders.

### 3.5. TNF

Mugnier et al. [61] evaluated whether the *TNF*-α promoter −308G/A polymorphism affects the response to IFX treatment using DAS28 in 59 RA patients. After 22 weeks of IFX treatment, in 42% of RA patients in the A/A or A/G groups and 81% of RA patients in the G/G group, DAS28 showed significant improvements to 1.24 ± 1.74 and 2.29 ± 1.33, respectively (*p* = 0.029). 

Cuchacovich et al. [63] investigated the effect of a *TNF*-α promoter polymorphism (G/A and G/G) circulating TNF-α levels on the IFX clinical response in 132 patients with RA. They found that serum TNF-α levels increased in patients who progressively showed significant improvement in all parameters with IFX treatment. This increase in TNF-α was a result of quantification of both free and circulating TNF-α and immune complexes of TNF-α bound to the anti-TNF-α monoclonal antibody [83]. When the two groups were analyzed separately, they found a statistically significant correlation between the ACR50 improvement criteria and the increase in TNF-α levels over time only in RA patients from the G/A group (*p* < 0.03).

Fonseca et al. [64] conducted a study of 22 RA patients to investigate the effect of the polymorphism at position −308 of the *TNF*-α gene on IFX treatment. Of all patients, 68% (*n* = 15) had the −308 GG genotype and 32% (*n* = 7) had the −308 AG genotype. After treatment with IFX for approximately 25 months, the DAS28 score of −308 GG genotype patients decreased, while the DAS28 score of −308 AG genotype patients slightly increased (*p* < 0.01). The Health Assessment Questionnaire was more evolved in the GG genotype group compared to that in the −308 AG group (*p* = 0.064). 

Marotte et al. [67] studied the association between the *TNF*-α −308 polymorphism and clinical response to IFX treatment in 198 patients with RA. They found that the *TNF*-α −308 polymorphism was not associated with the ACR response to IFX. The circulating *TNF*-α bioactivity level was higher in the A/A or A/G genotype group compared to that of the G/G genotype group. However, the difference in TNF-α protein level according to the genotype was not confirmed.

Chatzikyriakidou et al. [70] conducted a study on the correlation between the *TNFR1* gene polymorphism 36A/G, the *TNF*-α gene polymorphism −857C/T, −308G/A, −238G/A, and 489G/A, and the therapeutic effect in 27 RA patients treated with IFX. However, independent polymorphisms were not predictive of patient response to anti-TNF-α therapy.

Maxwell et al. [71] investigated the association between the response to infliximab by eight SNPs and DAS28 in the *TNF* gene region. The *TNF* −308 gene polymorphism was not related to the clinical IFX response, but it was negatively related to the *TNF* −238 G/A polymorphism (*p* = 0.028).

Fabris et al. [80] conducted a study to investigate whether the −238 G/G or +489 A/A *TNF*-α genotype in 66 patients with severe RA who received IFX differed from patients with mild-moderate RA. Patients with severe RA had a −238 G/G genotype in 100% of cases, whereas 92.8% of patients with mild-to-moderate RA and 92.5% of healthy individuals had a −238 G/G genotype (OR = 11.7; CI_95%_ = 0.6–216; *p* = 0.03). The +489 A/A genotype was less frequent in patients than in the control group (OR = 4.2; CI_95%_ = 0.97–18.4; *p* = 0.045). The −238 A/G genotype did not occur in patients with severe RA, and the mild-to-moderate RA genotype had the same frequency as the control group. Thus, −238 G/G homozygosity is associated with severe RA and the +489 A/A genotype may protect against poor RA outcomes.

### 3.6. TNFR1 and TNFR2

As mentioned earlier, Chatzikyriakidou et al. [70] reported that five independent polymorphisms were not predictive of patient response to IFX treatment. However, when they performed complex genotyping of both *TNFR2* and *TNF*-α gene polymorphisms, they found a statistically significant difference between good and poor IFX responders in the genotype association distribution of 676T/G and −857C/T (*p* = 0.008). Good responders more frequently carried the *TNFR2* allele 676T in homozygosity, with homozygosity of the *TNF*-α allele −857C/T compared with poor responders. The combination of 676T/G (*TNFR2*) and −857C/T (*TNF*-α) can be used to predict the efficacy of infliximab treatment.

Swierkot et al. [81] evaluated if five SNPs within the TNF-α and TNF receptor encoding genes (TNFA: G−308A, G−238A, C−857T; TNFR1A G36A; and TNFR1B T676G) affect the efficacy of TNF-α inhibitor therapy, including IFX, in 280 patients with RA. At 24 weeks of treatment, 45% of all patients achieved low disease activity or remission. After six months, lower disease activity or remission was observed more frequently in patients homozygous for the *TNFR1A* 36 allele than in patients homozygous for GG (52% versus 34%, *p* = 0.04) Additionally, at 24 weeks of treatment, the subgroup of RA patients homozygous for the *TNFA*-856T variant had a significantly lower DAS28 score compared to RA patients carrying the C allele (*p* = 0.045). No other polymorphisms were associated with EULAR responses at 12 and 24 weeks of treatment. In conclusion, homozygosity for the *TNFR1A* 36A allele and of *TNFA* −857T variant was associated with better response to anti-TNF therapy, including IFX.

## 4. Rituximab

B-cell targeting was first proposed as an RA treatment method in the 1990s based on the hypothesis that autoantibodies, such as rheumatoid factor, promote the survival of B-cells and thereby propagate chronic inflammation [84]. In addition, they can act as antigen-presenting cells through interaction with T-cells, which can stimulate inflammation [85]. Synovial B-cells are mainly part of the B-cell–T-cell aggregates that are closely related to the expression of factors such as A proliferation-inducing ligand (APPRIL), B-cell-activating factor (BAFF), and chemokines [86]. These molecules are key components of humoral adaptive immunity and are potential therapeutic targets for RA [87].

Rituximab (RTX) is a chimeric mAb with a specific affinity for CD20, which is expressed on most malignant B-cells [88]. It is approved to treat blood B-cell malignancies and non-hematologic B-cell-mediated diseases such as RA [89,90]. RTX binds to CD20 via a crystallizable fragment (Fc) and is reorganized in lipid rafts [91]. Thereafter, antibody-dependent cell-mediated cytotoxicity occurs due to the interaction of the Fcγ receptor expressed on the surface of effector cells (macrophages, granulocytes, and natural killer cells) with the Fc portion of RTX [92]. RTX in vivo mainly acts through immune-mediated mechanisms, including complement-dependent cytotoxicity involving NK cells and phagocytosis by macrophages and neutrophils [93,94]. The mechanisms of action of rituximab are only partly understood [95]. Table 3 described the genetic polymorphisms associated with RTX response.

### 4.1. FCGR2A and FCGR3

Jiménez Morales et al. [96] aimed to evaluate the effects of *FCGR2A* rs1801274 and FCGR3A rs396991 gene polymorphisms on response to RTX, EULAR response, remission, low disease activity (LDA), and DAS28 improvement in 55 patients diagnosed with RA and treated with RTX for 6, 12, and 18 months. Results showed that patients receiving RTX and carrying the T allele for *FCGR2A* rs1801274 gene polymorphism had a higher EULAR response at 6 months (OR = 4.86; CI_95%_ = 1.11–21.12; *p* = 0.035), 12 months (OR = 4.66; CI_95%_ = 0.90–24.12; *p* = 0.066) and 18 months (OR = 2.48; CI_95%_ = 0.35–17.31; *p* = 0.357), a higher remission at 6 months (OR = 10.625; CI_95%_ = 1.07–105.47; *p* = 0.044), and a higher improvement in DAS28 at 12 months (B = 0.782; CI_95%_ = −0.15–1.71; *p* = 0.098) and 18 months (B = 1.414; CI_95%_ = 0.19–2.63; *p* = 0.025). In addition, patients carrying the *FCGR3A* rs396991-G allele and receiving RTX had improved LDA (OR = 4.904; CI_95%_ = 0.84–28.48; *p* = 0.077) and DAS28 (B = −1.083; CI_95%_ = −1.98–−0.18; *p* = 0.021) at 18 months. These results suggest that the *FCGR2A* rs1801274 and *FCGR3A* rs396991 gene polymorphisms are good predictors of response to RTX treatment.

Quartuccio et al. [97] studied whether the *FCGR3A* −158 V/F polymorphism could affect the response to RTX in 212 RA patients. The *FCGR3A* genotype was not associated with a good/moderate EULAR response after four months of RTX treatment (*p* = 0.09). However, a significant difference between the VV and VF or FF genotypes was associated with a good/moderate EULAR response after six months of RTX treatment (*p* = 0.015 and *p* = 0.018, respectively), but not between the VF and FF genotypes (*p* = 0.96). RA patients with the VV genotype were associated with a good/moderate EULAR response after six months of RTX treatment by univariate logistic regression analysis (OR = 4.4; CI_95%_ = 1.4–13.5; *p* = 0.01).

Ruyssen-Witrand et al. [98] evaluated the association between single nucleotide polymorphisms in the *FCGR3A* gene and response to RTX treatment in 111 patients with RA who did not respond to or tolerate anti-TNF therapy. Of all RA patients, 81% (*n* = 90) had a response, 27% (*n* = 30) of which had a good response. Patients with RA carrying the *FCGR3A* −158V allele showed a statistically significant association with a higher response rate (OR = 4.6; CI_95%_ = 1.5–13.6; *p* = 0.006). In multivariate analysis, V allele retention was independently associated with response to RTX (OR = 3.8; CI_95%_ = 1.2–11.7; *p* = 0.023).

Similarly, Pál et al. [99] assessed the relationship between the *FCGR3A* polymorphism and the treatment outcome of RTX therapy in 52 RA patients. The distribution of *FCGR3A* genotypes was: VV: 15% (*n* = 8); VF: 65% (*n* = 34), FF: 19% (*n* = 10). A DAS28 reduction was shown in RA patients with three *FCGR3A* genotypes (VV: 1.98 ± 0.54, *p* = 0.008; VF: 2.07 ± 0.23, *p* < 0.001; FF: 1.59 ± 0.52, *p* = 0.014). Significant differences in DAS28 reduction on RTX treatment were identified between the VF heterozygote and the FF homozygote (*p* = 0.032) and between the heterozygote and VV+FF homozygotes (*p* = 0.017). Moreover, VV and VF patients achieved significant LDA compared to FF patients (VV: 62.5%; VF: 64.7%; FF: 30%).

However, conflicting results have been reported. Sarsour et al. [100] also conducted a study to determine if the *FCGR3A* polymorphism was associated with RTX efficacy in patients with RA. Longitudinal patient outcomes were assessed using the Clinical Disease Activity Index (CDAI) in 158 RTX-treated and 390 RA-treated TNF-α antagonists as controls. Similar changes in CDAI were observed for the three *FCGR3A* genotypes in the RTX-treated (VV: 4.56; VF: 7.44; FF: 4.75; *p* > 0.05) and TNF-α antagonist-treated patients (VV: 5.12; VF: 6.77; FF: 4.36; *p* > 0.05). The *FCGR3A* genotype was not significantly associated with treatment response in RTX-treated patients compared to TNF-α antagonist-treated patients (*p* = 0.86).

### 4.2. BAFF

Ruyssen-Witrand et al. [101] also determined whether *BAFF* polymorphisms were correlated with response to RTX treatment in 115 patients with RA. After 24 weeks of RTX treatment, the *BAFF* −871 C/C genotype was associated with a higher EULAR response rate than the T/T genotype (OR = 6.9; CI_95%_ = 1.6–29.6; *p* = 0.03). In the multivariate analysis, the C allele was independently related to the response to RTX (OR = 4.1; CI_95%_ = 1.3–12.7; *p* = 0.017).

### 4.3. IL-6

Fabris et al. [102] evaluated the effect of *IL-6* −174 G/C polymorphism on response to RTX. Treatment response was assessed using both EULAR and ACR criteria after six months of RTX treatment in 142 RA patients. According to the EULAR criteria, patients with the *IL-6* −174 C/C genotype showed less of a response to RTX than those with the GC/CC genotype (OR = 3.196; CI_95%_ = 1.204–8.485; *p* = 0.0234), and similar results were found when evaluating the response based on the ACR criteria.

### 4.4. TGFβ1

Daïen et al. [103] conducted a study on the association between the *TGFβ1* SNPs and responsiveness to RTX in 63 RA patients. Of these, 44 patients were defined as responders and 19 as non-responders. Both *TGFβ1-10* (rs1800470) and *TGFβ1-25* (rs1800471) were associated with clinical responses (OR = 1.6; CI_95%_ = 1.2–2.3; *p* = 0.002 and OR = 1.6; CI_95%_ = 1.3–1.9; *p* = 0.025, respectively). Additionally, the combination of the two SNPs elicited a much better RTX response (OR = 2.6; CI_95%_ = 1.4–4.6; *p* = 0.008). In addition, they researched the association between the RTX clinical response and genes coding for cytokines involved in synovitis (*IL-10*: rs1800896; *LTA*: rs909253 and rs1041981; *TNF*-α: rs1800629, rs80267959, and rs1799724; *TNFR2*: rs1061622) and genes related to RA susceptibility (*TRAF1*: rs1081848; *STAT4*: rs7574865; *TNFAIP3*: rs6920220, and *PTPN22L*: rs2476601), but no associations were noted.

## 5. Tocilizumab

IL-6 plays a pivotal role in the aforementioned interaction between B-cells and T-cells. It promotes differentiation of Ig-producing plasma cells, leading to hypergammaglobulinemia, associated with chronic inflammation, and also contributes to the sustained maintenance of B-cell subpopulation plasmablasts, known as precursors of plasma cells [104]. These surviving plasmablasts are a source of autoantibodies, such as anti-citrullinated protein/peptide antibodies, and may accompany related autoimmune and chronic inflammatory diseases [105].

Tocilizumab (TCZ) is a humanized monoclonal antibody that acts as an IL-6R antagonist [106]. Potential immunological effects of TCZ include induction and expansion of B-regulatory cells, decreased expression of pro-inflammatory cytokines and chemokine genes, and increased expression of healing genes in synovial fluid [107]. It is approved for use in juvenile idiopathic polyarthritis and giant cell arteritis [108]. TCZ may be used as monotherapy in the treatment of adult patients with moderate-to-severe active RA who have had an inadequate response to one or more DMARDs or TNF-α inhibitors [109]. We show the genes related to the response of RA to tocilizumab in Table 4.

### 5.1. FCGR2A and FCGR3A

Jiménez Morales et al. [96] evaluated the effects of *FCGR2A* rs1801274 and *FCGR3A* rs396991 gene polymorphisms on response to TCZ. A retrospective prospective cohort study was conducted on 87 patients with RA who received TCZ treatment for 6, 12, and 18 months. No association was found between the *FCGR2A* rs1801274 gene polymorphism and response to TCZ. In contrast, patients carrying the *FCGR3A* rs39699-TT genotype had a higher EULAR response (OR = 5.075; CI_95%_ = 1.20–21.33; *p* = 0.027) at 12 months. Therefore, patients carrying the genotype TT for rs1801274 *FCGR3A* would be better candidates for TCZ treatment.

### 5.2. HLA-DRB1

The *human leukocyte antigen (HLA)-DRB1* is the most strongly known genetic risk factor for the complex genetic etiology of RA, a group of alleles referred to as the shared epitope [112]. Wang et al. [110] performed the first genome-wide association study to identify genetic factors related to TCZ response of 1,683 patients with RA in six clinical studies. They identified putative associations with eight loci (rs11052877, rs4910008, rs9594987, rs10108210, rs703297, rs703505, rs1560011, and rs7055107) previously involved as risk alleles for RA or not linked to responses to other therapies. None of these polymorphisms are clearly associated with the IL-6 pathway, and there is no association between *HLA-DRB1* (shared epitope) with TCZ response.

### 5.3. IL-6R

Maldonado-Montoro et al. [111] examined the clinical parameters and rs12083537, rs2228145, rs4329505, rs11265618 gene polymorphisms of *IL6R* on TCZ EULAR response, LDA, and DAS28 improvement. They investigated a historic cohort of 77 patients with RA who had been treated with TCZ for 12 months. Of all gene polymorphisms, none were associated with EULAR response, remission, and DAS28 improvement. The AA genotype for rs12083537 (OR = 1.4; CI_95%_ = 1.13–2.01; *p* = 0.021) and the CC genotype for rs11265618 (OR = 1.3; CI_95%_ = 1.13–1.77; *p* = 0.031) are predictors of good response LDA. As a result of multivariate analysis, the AA genotype for rs12083537 (OR = 13.0; CI_95%_ = 2.31–72.91; *p* = 0.004) and the CC genotype for rs11265618 (OR = 12.15; CI_95%_ = 2.18–67.81; *p* = 0.004) were indicated. In conclusion, the *IL6R* polymorphisms AA genotype for rs12083537 and CC genotype for rs11265618 were significantly associated with higher LDA rates.

## 6. Newer Monoclonal Antibody Drugs and Genetic Polymorphisms of Their Targets

To provide precision medicine to individuals with a better understanding of the pathophysiology of RA, novel monoclonal antibody drugs that can treat RA through various mechanisms are being developed [113]. The association between various SNPs and response to biologic therapy in RA has been reported in several pharmacological studies [114]. Studies on the association of genetic polymorphisms with new targets and susceptibility to RA are being actively reported (Table 5).

IL-1, the first interleukin identified, is a major proinflammatory cytokine and a known pathogenic factor of auto-inflammation, auto-immunity, or infection. It is mainly secreted by macrophages, monocytes, and dendritic cells [156,157]. Canakinumab is a fully human anti-IL-1β monoclonal antibody drug that selectively neutralizes the bioactivity of IL-1β [158]. Another IL-11β monoclonal antibody, gevokizumab, was also studied in a phase lla study [159].

IL-17 is a pro-inflammatory cytokine mainly secreted by T helper 17 (Th17) cells and other T-cells [160]. This family includes six members from IL-17A to IL-17F [161]. IL-17 induces activation of the nuclear factor-κB, mitogen-activated protein kinases pathways, and the phosphoinositide-3 kinase pathway, leading to many inflammatory genes, mainly neutrophil-specific chemokines [162]. An excess of IL-17, which plays an important role in host defense, is observed in many chronic inflammatory and autoimmune diseases, including RA. It also contributes to tissue destruction [163]. Secukinumab and ixekizumab are humanized monoclonal antibody drugs against IL-17A, and brodalumab is a human immunoglobulin G2 monoclonal antibody drug against IL-17R. Several studies have reported that they are effective in treating RA when administered to patients who have no experience in biologic therapy or are resistant to anti-TNF therapy [164,165,166,167]. Recently, it has been reported that bimekizumab, developed for the dual blockade of IL-17A and IL-17F, effectively treats RA patients with an inadequate response to certolizumab pegol [168].

IL-23, a member of the IL-12 cytokine family, is a heterodimeric cytokine composed of an IL-23 p19 subunit and an IL-12/30 p40 subunit. It is mainly secreted by activated macrophages or dendritic cells [169]. They are known to induce differentiation of Th0 cells into Th17 cells and stimulate the production of pro-inflammatory cytokines such as TNF-α, IL-1β, IL-21, and IL-17 [170]. Ustekinumab is a human IgG1 monoclonal antibody drug that binds to the p40 subunit shared with IL-12 and IL-23 and blocks IL-12 and IL-23 signaling pathways through the inhibition of IL-12Rβ1 binding [171]. Guselkumab is the first IL-23 inhibitor approved for the treatment of severe plaque psoriasis by selectively targeting IL-23 [172]. However, the administration of both drugs to RA patients who did not respond to methotrexate treatment did not result in significant clinical improvement compared to the control group [173].

Granulocyte-macrophage colony-stimulating factor (GM-CSF) belongs to the colony-stimulating factor family of hematopoietic growth factors and is mainly produced by T-cells and stromal cells [174]. They are essential for regulating the function of mature bone marrow cells, such as macrophages, and induce the expression of HLA class Il antigens in synovial cells of RA patients [175]. Drugs targeting cytokines such as lenzilumab, namilumab, otilimab, and gimsilumab are being developed to block the GM-CSF pathway [176]. In particular, mavrilimumab, a human IgG4 monoclonal antibody against GM-CSF receptor α, showed an excellent clinical response and safety profile in RA patients [177].

The receptor activator of nuclear factor kappa B (RANKL) ligand belongs to the TNF superfamily and is an activated T-cell generator that regulates dendritic cell survival. Subsequent studies have reported that it is essential for osteoclast development [178,179]. Denosumab is a fully human anti-RANKL monoclonal antibody that competitively suppresses RANKL-RANK binding to inhibit osteoclast formation [180].

## 7. Conclusions and Future Challenges

Although the exact etiology of RA is still unknown, many researchers emphasize that it is caused by a combination of genetic and environmental factors. Several novel monoclonal antibodies that specify a target through various mechanisms have been developed and are showing excellent effects in the treatment of RA. In addition, recent review papers on sex and gender differences in RA treatment [181] and on different types of oral microbes involved in inducing RA progression [182] have suggested the possibility of more precise customization of treatment for individual patients. However, pharmacogenomic studies on the association between the new monoclonal antibody drugs and various genetic polymorphisms are insufficient. Moreover, factors involved in immunity and inflammation are very diverse, and there is currently no clear direction for personalized medicine [183]. Therefore, further research is essential, and this will increase the safety and efficacy of the newly developed monoclonal antibody drugs, enabling more complete precision medicine. This review reveals the complex genetic basis for the response to monoclonal antibody drugs among biological DMARDs used to treat RA, and it may contribute to important advances in understanding the molecular mechanisms involved in these therapies.

## Figures and Tables

**Table 1:** Genetic polymorphisms known to affect adalimumab response in patients with rheumatoid arthritis (RA).

Biological Agent	Gene	Polymorphism	Clinical Outcome(s)	Refs.
Adalimumab	*FCGR2A*	rs1801274	Significantly associated with the clinical response	[39]
	*DHX32*	rs12356233		
	*RGS12*	rs4690093	Nominally significantly associated with the response	
	*IL-6*	rs1800795 (−174 G/C)	Significantly associated with a better response	[40]
	*PTPN22*	1858 C/T	No effect on efficacy adalimumab	[41]
	*TNF*	rs1800629 (−308 G/A)	*TNF*-308 G/G genotype associated with better clinical effect than *TNF* −308 A/G	[42,43,44,45]
		−238A/G, −308A/G and −857C/T	*TNF*-α locus haplotype (−238G/−308G/−857C) was associated with a lower response	[46]
	*TNFR2*	676 T/G	*TNFR2* 676 T/T genotype associated with a better clinical response than *TNFR2* 676 T/G	[47]

*DHX32*, DEAH (Asp-Glu-Ala-His)-box polypeptide 32; *FCGR2A*, Fc fragment of immunoglobulin G receptor IIa; *IL-6*, interleukin 6; *PTPN22*, protein tyrosine phosphatase non-receptor type 22; *RGS12*, regulator of G protein signaling 12; *TNF*, tumor necrosis factor; *TNFR2*, tumor necrosis factor receptor 2.

**Table 2:** Genetic polymorphisms known to affect infliximab response in patients with RA.

Biological Agent	Gene	Polymorphism	Clinical Outcome(s)	Refs.
Infliximab	*FCGR2A*	rs1801274	No effect on response to infliximab	[39]
		H131R	*FCGR2A*-RR was associated with a better response compared to RH or HH	[78]
	*FCGR3A*	V158F	*FCGR3A*-FF was associated with a better response compared to VV or VF	
	*RGS12*	rs2857859	Nominally significantly associated with the response	[39]
	*PTPN22*	1858 C/T	No effect on response to infliximab	[41]
	*MHC*	MHC polymorphisms	TNFa11;b4 associated with responders	[79]
	*TNF*	rs1800629 (−308 G/A)	*TNF*-308 G/G associated with a better response than *TNF* −308 A/A or A/G	[61,63,64]
		−308 G/A	No effect on response to infliximab	[67]
		−308 G/A, −238 G/A, 489 G/A, −857 C/T		[70]
		rs361525 (−238 G/A)	*TNF* −238G/A was associated with poorer response	[71]
		−238 G/G, +489 A/A	*TNF* −238 G/G was associated with severe RA	[80]
		−308 G/A, −238 G/A	No effect on response to infliximab	[81]
	*TNFR1*	36 A/A, 676 T/G	*TNFR1A* 36 A/A was associated with better response compared to G/G and *TNFR1B* 676 T/G was not associated with response to infliximab	
		36 A/G	No effect on response to infliximab	[70]
	*TNFR2*	676 T/G	A combination of 676 T/G (*TNFR2*) and −857 C/T (*TNF*-α) could be used for prognosis of clinical response to infliximab	

*FCGR2A*, Fc fragment of immunoglobulin G receptor IIa; *FCGR3A*, Fc fragment of immunoglobulin G receptor IIIa; *MHC*, major histocompatibility complex; *PTPN22*, protein tyrosine phosphatase non-receptor type 22; *RGS12*, regulator of G protein signaling 12; *TNF*, tumor necrosis factor; *TNFR1*, tumor necrosis factor receptor 1; *TNFR2*, tumor necrosis factor receptor 2.

**Table 3:** Genetic polymorphisms known to affect rituximab response in patients with RA.

Biological Agent	Gene	Polymorphism	Clinical Outcome(s)	Refs.
Rituximab	*FCGR2A*	rs1801274	*FCGR2A* rs1801274-T/T genotype was associated with better clinical response to rituximab	[96]
	*FCGR3A*	rs396991	*FCGR3A* rs396991-G allele genotype was associated with better response to rituximab	
		−158 V/F	*FCGR3A* −158 V/V was associated with a better response than V/F or F/F	[97]
			*FCGR3A* −158 V allele was independently associated with better response to rituximab	[98]
			*FCGR3A* −158 V/V and V/F was associated with a better response than F/F	[99]
			*FCGR3A* −158 V/F was not associated with CDAI response to rituximab	[100]
	*BAFF*	−871 C/T	*BAFF* −871 C/C was associated with a better response than T/T	[101]
	*IL-6*	rs1800795 (−174 G/C)	*IL-6* −174 GG/GC genotypes was associated with better responses than with −174 C/C genotypes	[102]
	*TGFβ1*	rs1800470, rs1800471	*TGFβ1* SNPs was associated with good response to rituximab	[103]

*BAFF*, B-cell-activating factor; *CDAI*, Clinical Disease Activity Index; *FCGR2A*, Fc fragment of immunoglobulin G receptor IIa; *FCGR3A*, Fc fragment of immunoglobulin G receptor IIIa; *IL-6*, interleukin 6; *SNP*, single nucleotide polymorphism; *TGFβ1*, Transforming growth factor beta-1.

**Table 4:** Genetic polymorphisms known to affect tocilizumab response in patients with RA.

Biological Agent	Gene	Polymorphism	Clinical Outcome(s)	Refs.
Tocilizumab	*FCGR2A*	rs1801274	Not associated with response to tocilizumab	[96]
	*FCGR3A*	rs396991	*FCGR3A* rs396991-T/T genotype is associated with better EULAR response to tocilizumab	
	*HLA-DRB1*	rs11052877, rs4910008, rs9594987, rs10108210, rs703297, rs703505, rs1560011, rs7055107	The shared epitope *HLA-DRB1* had no association with tocilizumab response	[110]
	*IL-6R*	rs12083537, rs2228145, rs4329505, rs11265618	rs12083537-A/A and rs11265618-C/C were associated with higher LDA rates	[111]

*EULAR*, European League Against Rheumatism; *FCGR2A*, Fc fragment of immunoglobulin G receptor IIa; *FCGR3A*, Fc fragment of immunoglobulin G receptor IIIa; *HLA-DRB1*, human leukocyte antigen-beta chain 1; *IL-6R*, interleukin 6 receptor; *LDA*, low disease activity.

**Table 5:** Newer monoclonal antibody drugs and genetic polymorphisms of their targets.

Biological Agent	Polymorphism	Clinical Outcome(s)	Refs.
Canakinumab, Gevokizumab	*IL-1α*	rs17561	[115]
	*IL-1β*	rs16944	[115]
		rs16944, rs1143634	[116]
			[117]
			[118]
			[119]
	*IL-1Ra* (*IL-1RN*)	2018 C/T	[117]
		5111 T/C, 2017 T/C	[115]
Secukinumab, Ixekizumab, Bimekizumab	*IL-17A*	rs4711998, rs8193036, rs3819024, rs2275913, rs7747909	[120]
		rs2275913, rs3804513, rs3748067, rs1974226	[121]
		rs2275913	[122]
			[123]
		rs2275913, rs3819024, rs3819025, rs4711998, rs8193036, rs8193037, rs3804513	[124]
		rs2275913, rs3804513	[125]
		rs2275913	[126]
			[127]
			[128]
			[129]
			[130]
			[131]
		rs2275913, rs3819024, rs4711998, rs8193036	[132]
		rs2275913	[133]
			[134]
			[135]
			[136]
			[137]
			[138]
	*IL-17F*	rs763780, rs2397084	[121]
		rs763780	[122]
		rs763780, rs2397084	[123]
		rs763780	[125]
			[126]
		rs763780, rs11465553, rs2397084	[127]
		rs763780, rs2397084	[128]
			[129]
		rs763780	[130]
		rs763780, rs2397084	[132]
			[134]
		rs763780	[135]
		rs763780, rs2397084	[136]
		rs763780	[138]
Brodalumab	*IL-17RC*	rs708567	[133]
Ustekinumab, Guselkumab	*IL-23R*	rs11209026, rs134315, rs10489629, rs7517847	[125]
		rs11209026, rs1343151, rs10489629	[128]
		rs10889677	[130]
		rs1004819, rs10489629, rs11209026, rs1343151, rs10889677, rs11209032, rs1495965	[139]
		rs10889677, rs2201841, rs1884444	[140]
		rs1004819, rs7517847, rs10489629, rs2201841, rs1343151, rs11209032, rs1495965	[141]
		rs11209026, rs2201841, rs10889677	[142]
Lenzilumab, Namilumab, Otilimab, Gimsilumab	*GM-CSF*	177 T/C	[143]
		545 G/A, 3606 T/C, 3928 C/T	[144]
		677 C/C	[145]
		3606 T/C, 3928 C/T	[146]
		677 A/C	[147]
		3928 C/T, 3606 T/C	[148]
Denosumab	*RANKL*	rs9533156, rs2277438, rs1054016	[149]
		rs2277438	[150]
		rs2277438, rs7984870	[151]
		rs2277438, rs9533156	[152]
		rs7325635, rs7988338	[153]
			[154]
		rs2277438, rs9533156	[155]

*IL-1α*, interleukin-1 alpha; *IL-1β*, interleukin-1 beta; *IL-1Ra* (or *IL-1RN*), interleukin-1 receptor antagonist; *IL-17A*, interleukin-17A; *IL-17F*, interleukin-17F; *IL-17RC*, interleukin-17 receptor C; *IL-23*, interleukin-23 receptor; *GM-CSF*, granulocyte-macrophage colony-stimulating factor; *RANKL*, Receptor activator of nuclear factors κB ligand.

## Data Availability

Not applicable.
